# Hepatic Candidiasis in an Immunocompetent Patient: A Diagnostic Challenge

**DOI:** 10.7759/cureus.13935

**Published:** 2021-03-16

**Authors:** Zorays Moazzam, Amman Yousaf, Zahid Iqbal, Ahmad Tayyab, Muhammad Hashim Hayat

**Affiliations:** 1 Surgery, Aga Khan University Hospital, Karachi, PAK; 2 Radiology, Hamad General Hospital, Doha, QAT; 3 Radiology, Services Hospital, Lahore, PAK; 4 Internal Medicine, Services Hospital, Lahore, PAK; 5 Medicine, Vanderbilt University Medical Center, Nashville, USA

**Keywords:** hepatic abscess, candidiasis, immunocompetent, computed tomography abdomen

## Abstract

Hepatic candidiasis is a manifestation of disseminated candidiasis, which typically presents in immunocompromised patients. Focal hepatic candidiasis in immunocompetent patients, however, is infrequent/extremely rare. We present the case of an immunocompetent female patient who presented with respiratory distress and right-sided pleural effusion. The pleural fluid tap did not grow anything, and a contrast-enhanced computed tomography (CT) scan revealed a right liver lobe subcapsular collection. CT-guided aspiration and culture resulted in Candida albicans growth. The patient responded to oral fluconazole, and a follow-up CT scan demonstrated resolution of the collection. Although hepatic candidiasis rarely occurs in immunocompetent patients, it should be included in the differential diagnosis of hepatic abscesses, as timely diagnosis and management are crucial in conferring a good prognosis.

## Introduction

Candida is a commensal yeast found predominantly in the orogastrointestinal tract [1]. Chronic disseminated candidiasis is a form of invasive candidiasis, which most commonly affects the liver and spleen. It typically presents in neutropenic patients with hematologic malignancies and receiving chemotherapy [2]. However, fungal infections account for <2% of the isolates in hepatic abscesses in immunocompetent patients [3]. Non-specific clinical presentations and low blood culture yield to identify candida make the diagnosis a challenging prospect [4,5]. In this article, we present a case of a female patient who presented with respiratory distress and abdominal pain as symptoms of hepatic candidiasis.

## Case presentation

A 68-year-old female patient with a past medical history of hypertension and chronic kidney disease (mild, stage 2 with glomerular filtration rate [GFR] of 75 mL/min) presented to the emergency department with a one-week history of dry cough and dyspnea. She had non-radiating, mild right upper quadrant abdominal pain and nausea without vomiting. Her presenting vitals were temperature 37.2 ºC, heart rate 85 beats/min, respiratory rate 18/min, and blood pressure 115/80 mmHg. On examination, there was mild right upper quadrant tenderness and bronchial breath sounds in the right lower lung fields. Laboratory tests showed leukocytosis of 34 x 109 cells/L with a neutrophilic predominance and an elevated C-reactive protein (CRP) of 311. Liver function tests were mildly elevated, and her blood culture revealed no growth.

**Figure 1:**
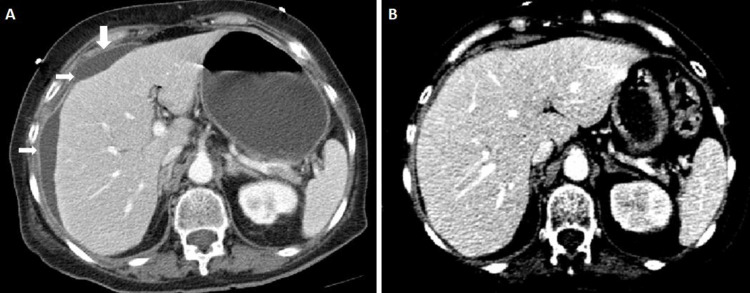
Contrast-Enhanced CT Scan Abdomen. (A) An axial selected section of the CT abdomen showing a hypodense fluid density collection in the subcapsular and perihepatic region of the liver, mainly in the right lobe (white arrows). (B) An axial selected section, from a follow-up CT scan abdomen showing the resolution of the previously mentioned collection. CT - computed tomography

The chest x-ray demonstrated an obscured right costophrenic angle suggestive of minimal pleural effusion. The diagnostic pleural tap demonstrated no growth; however, it had a white cell count of 1.7 x 109 cells/L with a neutrophilic predominance. Contrast-enhanced computed tomography (CT) abdomen denoted a hypodense subcapsular thick fluid density lesion in the liver's upper pole, mainly involving the right lobe of the liver and perihepatic region (Figure 1A).

Prophylactic antibiotic therapy for the pyogenic liver abscess was started, and it was decided to proceed with CT-guided drainage and abscess culture. The culture was positive for Candida albicans. The patient was prescribed oral fluconazole, and a follow-up contrast-enhanced CT scan showed resolution of the liver lesion or perihepatic fluid (Figure 1B). The patient improved clinically and has no recurrence of the signs and symptoms to date.

## Discussion

The etiology of liver abscesses is polymicrobial. A series of 233 patients had reported a fungal component in 22% of liver abscesses [6]. However, owing to the difficulty in diagnosis and severity of infection, it accounts for a disproportionately high mortality rate [7]. Hepatosplenic candidiasis (HSC) almost always occurs in patients with hematological malignancies, with an incidence of about 10%. Most commonly, HSC is seen in acute lymphocytic leukemia, followed by acute myeloid leukemia and lymphoma [3]. Hepatic involvement is frequently seen as part of invasive candidiasis in immunocompromised patients [7]. However, focal HSC has also been reported. HSC in patients with no risk factors is extremely rare [8].

To date, we could only identify three reports of HSC in immunocompetent patients. Gottlieb et al. reported an immunocompetent woman who developed Candidal hepatic abscesses as a result of pylephlebitis. However, the causative species was Candida dubliniensis in this case [9]. Menachery et al. and Hasan et al. both reported a solitary C. albicans liver abscess in their patients [10,11]. However, the patient reported by Hasan et al. developed the liver abscess primarily as a complication of repeated endoscopic retrograde cholangiopancreatography (ERCP) and subtotal cholecystectomy [11]. Furthermore, as in our patient, respiratory symptoms as the primary presenting complaint have not been documented before, and thus physicians should remain cognizant of this unique presentation.

Multiple identifiable factors might contribute to hepatic candidiasis' pathogenesis, including cytotoxic chemotherapeutic regimens, prolonged use of broad-spectrum antibiotics, neutropenic conditions, and gastrointestinal mucosal damage. Specifically, severe neutropenia, coupled with mucosal damage, can allow Candida species to deposit in the liver and spleen. Commonly observed symptoms include persistent fever, abdominal pain, loss of appetite, nausea, vomiting, dysphagia, and gastrointestinal bleeding [12]. The examination can reveal oropharyngeal candidiasis, abdominal distention with right upper quadrant pain, hepatomegaly, and rarely splenomegaly. Biochemical parameters are usually non-specific for HSC diagnosis. Nevertheless, a high serum alkaline phosphatase and bilirubin are almost always present, and inconsistent elevation in ALT and AST is also seen [7]. Moreover, blood cultures are negative in more than 50% of the cases [13].

Abdominal ultrasound and a contrast-enhanced CT scan of the abdomen are the initial investigations of choice. A contrast-enhanced CT scan is the most sensitive investigation, typically depicting multiple, round, hypoattenuating lesions with a central enhancement. A percutaneous liver biopsy can help the definitive diagnosis of Candida in almost three-quarters of the patients if performed [9]. A diagnostic laparoscopy revealing white lesions on the liver surface can also aid in diagnosis [14].

Despite the good prognosis of HSC in immunocompetent patients, a high index of clinical suspicion, repeat imaging, or liver biopsy is needed before initiating antifungal therapy, as in our case. Amphotericin B has been widely used as individual therapy or in combination with flucytosine [7]. Complete eradication of hepatic candidiasis is challenging, and prolonged therapy should be considered for better outcomes. Follow-up imaging with a contrast-enhanced CT is recommended every two to three months until the lesions' resolution or calcification is noted [15].

## Conclusions

From our case and the literature review, we conclude that hepatic candidiasis can rarely occur in immunocompetent patients without identifiable risk factors. Physicians should not exclude hepatic candidiasis in immunocompetent patients as a cause of the liver abscess. The diagnosis is challenging and might need repeated imaging and liver biopsy for confirmation. Prompt diagnosis and treatment confer a good prognosis.

## References

[1] Van De Veerdonk FL, Kullberg BJ, Netea MG (2010). Pathogenesis of invasive candidiasis. Curr Opin Crit Care.

[2] Chen CY, Cheng A, Tien FM, Lee PC, Tien HF, Sheng WH, Chen YC (2019). Chronic disseminated candidiasis manifesting as hepatosplenic abscesses among patients with hematological malignancies. BMC Infect Dis.

[3] Fiore M, Cascella M, Bimonte S, Maraolo AE, Gentile I, Schiavone V, Pace MC (2018). Liver fungal infections: an overview of the etiology and epidemiology in patients affected or not affected by oncohematologic malignancies. Infect Drug Resist.

[4] Albano D, Bosio G, Bertoli M, Petrilli G, Bertagna F (2016). Hepatosplenic candidiasis detected by (18)F-FDG-PET/CT. Asia Ocean J Nucl Med Biol.

[5] Cornely OA, Bangard C, Jaspers NI (2015). Hepatosplenic candidiasis. Clin Liver Dis.

[6] Huang CJ, Pitt HA, Lipsett PA, Osterman FA Jr, Lillemoe KD, Cameron JL, Zuidema GD (1996). Pyogenic hepatic abscess: changing trends over 42 years. Ann Surg.

[7] Tashjian LS, Abramson JS, Peacock JE Jr (1984). Focal hepatic candidiasis: a distinct clinical variant of candidiasis in immunocompromised patients. Rev Infect Dis.

[8] Spindel SJ, Darouiche RO, Saeed ZA (1996). Hepatosplenic candidiasis in non-neutropenic patients: a case report and literature survey. Int J Antimicrob Agents.

[9] Gottlieb LB, Hashemi A, Dewar R, Salama C (2019). Hepatic candidiasis in an immunocompetent woman: a rare complication of pylephlebitis. Infect Dis Clin Pract.

[10] Menachery J, Chawla Y, Chakrabarti A, Duseja A, Dhiman R, Kalra N (2012). Fungal liver abscess in an immunocompetent individual. Trop Gastroenterol.

[11] Hasan S, Fearn R (2018). Fungal liver abscess in an immunocompetent patient who underwent repeated ERCPs and subtotal cholecystectomy. BMJ Case Rep.

[12] Meunier F (1989). Candidiasis. Eur J Clin Microbiol Infect Dis.

[13] Thaler M, Pastakia B, Shawker TH, O’Leary T, Pizzo PA (1988). Hepatic candidiasis in cancer patients: the evolving picture of the syndrome. Ann Intern Med.

[14] Lewis JH, Patel HR, Zimmerman HJ (1982). The spectrum of hepatic candidiasis. Hepatology.

[15] Kauffman CA, Bradley SF, Ross SC, Weber DR (1991). Hepatosplenic candidiasis: successful treatment with fluconazole. Am J Med.

